# Racial Disparities in Climate Change-Related Health Effects in the United States

**DOI:** 10.1007/s40572-022-00360-w

**Published:** 2022-05-28

**Authors:** Alique G. Berberian, David J. X. Gonzalez, Lara J. Cushing

**Affiliations:** 1 Department of Environmental Health Sciences, University of California, 650 Charles E. Young Drive South, 71-259 CHS, Los Angeles, CA 90095 USA; 2 School of Public Health and Department of Environmental Science, Policy, and Management, University of California, Berkeley, CA USA

**Keywords:** Global warming, Environmental justice, Health disparities, Environmental racism, Health equity

## Abstract

**Purpose of Review:**

Climate change is causing warming over most parts of the USA and more extreme weather events. The health impacts of these changes are not experienced equally. We synthesize the recent evidence that climatic changes linked to global warming are having a disparate impact on the health of people of color, including children.

**Recent Findings:**

Multiple studies of heat, extreme cold, hurricanes, flooding, and wildfires find evidence that people of color, including Black, Latinx, Native American, Pacific Islander, and Asian communities are at higher risk of climate-related health impacts than Whites, although this is not always the case. Studies of adults have found evidence of racial disparities related to climatic changes with respect to mortality, respiratory and cardiovascular disease, mental health, and heat-related illness. Children are particularly vulnerable to the health impacts of climate change, and infants and children of color have experienced adverse perinatal outcomes, occupational heat stress, and increases in emergency department visits associated with extreme weather.

**Summary:**

The evidence strongly suggests climate change is an environmental injustice that is likely to exacerbate existing racial disparities across a broad range of health outcomes.

**Supplementary Information:**

The online version contains supplementary material available at 10.1007/s40572-022-00360-w.

## Introduction

Climate change is widely considered the greatest global health threat. However, its impacts are not borne equally. Environmental and climate justice advocates warn that US communities of color, Indigenous peoples, and low-income families and children are more vulnerable to changing climate conditions and have the least resources to protect against and recover from extreme weather events [1]. Racially and socioeconomically marginalized communities in the USA have been shown to experience greater impacts from storm and flood events, extreme heat, and infectious diseases that are becoming more frequent due to climate change [2, 3]. Children’s physiology and dependence on others for basic needs also make them particularly vulnerable. Collectively, this raises concerns that climate change will exacerbate existing social and economic inequalities and worsen racialized health disparities among both children and adults [4, 5].

In this scoping review, we summarize the recent (2017–2022) evidence that climatic changes are having a disparate impact on the health of people of color in the USA, including children. Attributing any particular event (e.g., cyclone, heat wave, wildfire) or health burden to anthropogenic climate change is challenging, although advancements in techniques for detection and attribution are making such analyses more possible [6, 7]. Given that few health studies have attempted detection or attribution, we considered research that assessed health impacts in relation to exposures whose occurrence and severity are increasing due to climate change, including temperature, extreme weather events, drought, and wildfire.

Health disparities refer to systematic, plausibly avoidable health differences that adversely affect racially and socioeconomically marginalized groups [8]. Here, we focus on racial/ethnic health disparities that disproportionately impact people of color who experience social marginalization due to systematic racism and the legacy of race-based discrimination in the USA. We define people of color as those identifying as Black, Hispanic/Latinx, Asian, Native American, Pacific Islander, or multiracial, while recognizing the variation in experience within these categories and the omission of important groups experiencing discrimination (e.g., Arabs who are often categorized as White), as well as the fluidity of race as a social construct over time.

Epidemiologists typically assess racial health disparities by examining the magnitude and statistical significance of stratum-specific effects or interaction terms between race and the exposure of interest. However, relying on this approach alone can fail to identify exposures that result in health disparities in cases where the exposure or health outcome are unequally distributed [9]. For example, even if the estimated effect on mental health of experiencing a flood was the same for Black and White individuals, extreme weather will result in a health disparity if racial segregation has concentrated Black communities in more flood-prone areas of a city and Black people therefore experience greater flooding than White people. It is with this framework in mind that we synthesize the recent literature.

## Methods

We considered peer-reviewed studies published from January 2017 to January 2022 that focused on US populations. Our inclusion criteria required studies to empirically examine disparities in a human health outcome related to a climate exposure by either (1) comparing two or more racial/ethnic groups or (2) focusing exclusively or primarily on people of color (e.g., studies based in Puerto Rico or focused on Latinx farmworkers). Categories of climate change exposure pathways and related health outcomes were adapted from the Intergovernmental Panel on Climate Change (IPCC) Fifth Assessment Report (2014) (Table 1) [10]. We did not consider studies that assessed health disparities related to changes in exposure to natural environment characteristics like green and blue space. While the research on health impacts and health disparities related to green and blue space has grown recently, there are limited longitudinal studies examining changes in green and blue space that could be associated with climate change, in part because their changes are slow to occur.

**Table 1:** Climate change exposure pathways and associated environmental and health impacts (adapted from the IPCC Fifth Assessment Report, 2014)

Climate change exposure pathways	Impacts
Climate- and weather-related impacts	Temperature, drought, sea level rise, flood, cyclone, hurricane, flood, wildfire, precipitation
Ecosystem-mediated impacts	Infectious disease (e.g., vector-, food- and water-borne), allergens
Human institution-mediated impacts	Occupational health, mental health, natural technological disaster, nutrition

Eligible studies were identified by a search of PubMed on January 28, 2022 that combined three separate keyword sets: climate-related terms (e.g., “temperature,” “precipitation,” “hurricane”), health impacts (e.g., “mortality,” “injury,” “perinatal”) and terms related to race, ethnicity, and disparities (e.g., “Black,” “Hispanic,” “environmental justice”) (Table S1). Records were screened for relevance based on their title and abstract. Select studies were assessed for eligibility by screening the full text, resulting in 85 studies. Four additional studies were then identified using backward and forward citation searches for a final sample of 89 studies.

Information on the climate exposure, health outcome, racial/ethnic groups, geographic scope, and findings were then extracted into a summary table. There was variation in how authors defined racial/ethnic groups. For coherence, we summarize findings using the following broad groupings: Asian, Black, Latinx (of any race), Native American, Pacific Islander, and White, although some studies included much more specific groups (e.g., non-Hispanic Black, Native Alaskan, Vietnamese), which we refer to when describing specific results.

## Disparities in Climate-Related Health Outcomes

The recent literature has focused on changing temperatures (including extreme heat and cold), and hurricane and flood events. Fewer studies examined wildfires, drought, and precipitation. The majority of studies on adult populations (Table 2) examined mortality, mental health, and morbidity outcomes like heat-related illnesses and cardiorespiratory diseases resulting in emergency department (ED) visits and hospitalizations. Most studies focused on children (Table 3) examined perinatal outcomes, including preterm birth and low birth weight.

**Table 2:** Studies on climate-related adult health outcomes included in review

Climate impact	Health outcome	Racial/ethnic group	Geographic scope	Example findings of disparities
Temperature	Mortality [11–15]	All	PA, PR, TN, US	Significantly increased risk in stroke and cardiovascular disease mortality in Puerto Rico during the summers of 2012 and 2013, the hottest on record, associated with elevated temperature [14]
	Mental health [16–19]	All	CA, NY, TN	Greatest increase in mental health-related ED visit risk in California (2005–2013) among Hispanics compared to Whites for every 10°F increase in mean daily temperature [16]
	Cardiovascular [20, 21]	B, L, W	FL, MD, US	Significantly higher odds of myocardial infarction hospitalization associated with extreme heat events among non-Hispanic Blacks than non-Hispanic Whites in Maryland from 2000 to 2012 [21]
	Renal illness [22–24]	All	MA, NY, PA, US	Higher risk of hospitalization and mortality among non-Hispanic Blacks with end-stage renal disease than non-Hispanic Whites associated with heat in Boston, Philadelphia, and New York City [24]
	Occupational [25–28]	B, L, W	FL, IA, US, WA	84% of Latinx farmworkers in a Florida prospective cohort experienced at least one heat-related illness symptom and 40% experienced three or more. Females had higher odds than males [27]
	ED visits, HA [29–38]	All	AZ, CA, FL, MI, US, VA	Higher risk for ED visits among groups of color identified as “non-white” than Whites due to cold weather conditions in Roanoke and Charlottesville, Virginia, from 2010 to 2017 [31]
	Respiratory [39–42]	B, L, W	GA, NE, US	More than three times higher ED asthma diagnosis risk among Blacks than non-Blacks associated with 2012 heatwave in Douglas County, Nebraska [39]
Hurricane, flood	Mortality [43–45, 46••]	L	PR	Estimated mortality rate of 14.3 deaths per 1,000 people from Hurricane Maria—a total of 4,645 excess deaths, which translates to a 62% increase in mortality rate compared to the prior year [46••]
	Mental health [47–55]	A, B, L, W	LA, NJ, NY, PR, TX	Highest risk for poor mental health among Hispanics compared to Blacks and Whites among Hurricane Sandy survivors in New York and New Jersey [50]
	Cardiovascular [56, 57]	B, W	LA	Higher likelihood for Blacks with PTSD 1–2 years after Hurricane Katrina to have a cardiovascular disease event than those without PTSD. No significant association among Whites [56]
	Reproductive [58, 59]	B, L, W	LA, NY	Highest increase in ED visits related to pregnancy complications among Hispanics associated with power outages after Hurricane Sandy in New York followed by Blacks and Whites [59]
	Chronic [60, 61]	L	PR	Increased prevalence of chronic disease (e.g., hypertension, high cholesterol) following Maria [60]
	Infectious [62]	B, L, N, W	NC	Higher risk of acute gastrointestinal illness ED visits among American Indians and Blacks compared to non-Hispanic Whites associated with Hurricane Florence [62]
	ED visits, HA [63–67]	B, L, W	NY, PR	68.9% increase in Hurricane Maria-related ED visits in NYC, a primary destination for displaced Puerto Ricans, during and after the hurricane’s landfall, as compared to 8 weeks prior [63]
Wildfire	ED visits, HA [68••, 69, 70]	B, N, W	AK, US, Western US	Higher odds of asthma- and heart failure-related ED visits among Alaska Natives than non-Alaska Natives associated with wildfire PM_2.5_ exposure during 2015–2019 wildfire seasons in Alaska [69]
Other	Mortality [71, 72••, 73••]	All	US	Highest mortality rate from natural hazards (e.g., heat/cold, storm) among Native Americans, followed by Blacks, Whites, Latinos, and Asians/Pacific Islanders among those aged 0–84 [71]
	Respiratory [74•]	B, W	MD	Higher risk of asthma hospitalization among Blacks than Whites associated with late vs. normal and very early vs. normal onset of Spring in Maryland from 2001 to 2012 [74•]
	Infectious [75–77]	All	MA, US	Higher risk for influenza ED visits among Blacks than Whites and those categorized as “other” race associated with extreme precipitation in Massachusetts [76]

*All* Asian, Black, Latinx, Native American, Pacific islander, White. *A* Asian, *B* Black, *L* Latinx, *N* Native American, *P* Pacific Islander, *W* White. *ED* Emergency Department, *HA* hospital admissions. Other category includes drought, precipitation, phenological events, multiple climate exposures

**Table 3:** Studies on climate-related children’s health outcomes included in review

Climate impact	Health outcome	Racial/ethnic group	Geographic scope	Example findings of disparities
Temperature	Perinatal [78, 79••, 80–84]	All	CA, MI, MN, NC, US	Greater likelihood of preterm birth associated with heat exposure among non-Hispanic Blacks and Hispanics than non-Hispanic Whites across 50 US metropolitan areas from 1981 to 1988 [81]
	Mortality [85–87]	B, L, W	FL, PA, US	Higher risk for sudden infant death syndrome (SIDS) among Black infants associated with a 10°F increase in temperature than Whites from 1972 to 2006 in 210 US cities [85]
	ED visit, HA [88•, 89–91]	All	GA, NY, US	Higher risk of all-cause ED visits (e.g., ear infection, behavioral disorder) among non-White than White children associated with extreme heat across 47 US children’s hospitals (2016–2018) [88•]
	Occupational [92•, 93, 94]	L	NC	45.5% of migrant and non-migrant child Latinx farmworkers in a prospective cohort experienced heat-related illness (e.g., dizziness, nausea, fainting) while working over the past year [94]
Hurricane, flood	Mental health [95]	L	PR	7.2% of Puerto Rico elementary school students (*n* = 6,900) had clinically significant symptoms of PTSD 5–9 months after Maria, with girls exceeding the clinical cutoff score more often than boys [95]
	Infant gut bacteria [96]	L	PR	Significantly decreased abundance of *Veillonella* gut bacteria among infants whose birth parent experienced food insecurity during pregnancy following Hurricane Maria [96]
	Body mass index [97]	A, B, L	T	Significantly greater body mass index (BMI) decrease among ethnic minority adolescents highly impacted by Hurricane Harvey compared to those less severely impacted [97]
Other	Perinatal [98, 99]	A, B, L, W	CA, PR	Elevated preterm birth risk in Ponce, San Juan, and Mayagüez, Puerto Rico, associated with precipitation and storm and flood event intensity and frequency from 1994 to 2012 [98]

*A* Asian, *B* Black, *L* Latinx, *N* Native American, *P* Pacific Islander, *W* White, *All* Asian, Black, Latinx, Native American, Pacific islander, White. *ED* Emergency Department, *HA* hospital admissions. Other category includes drought, precipitation, phenological events, multiple climate exposures

**Fig. 1:**
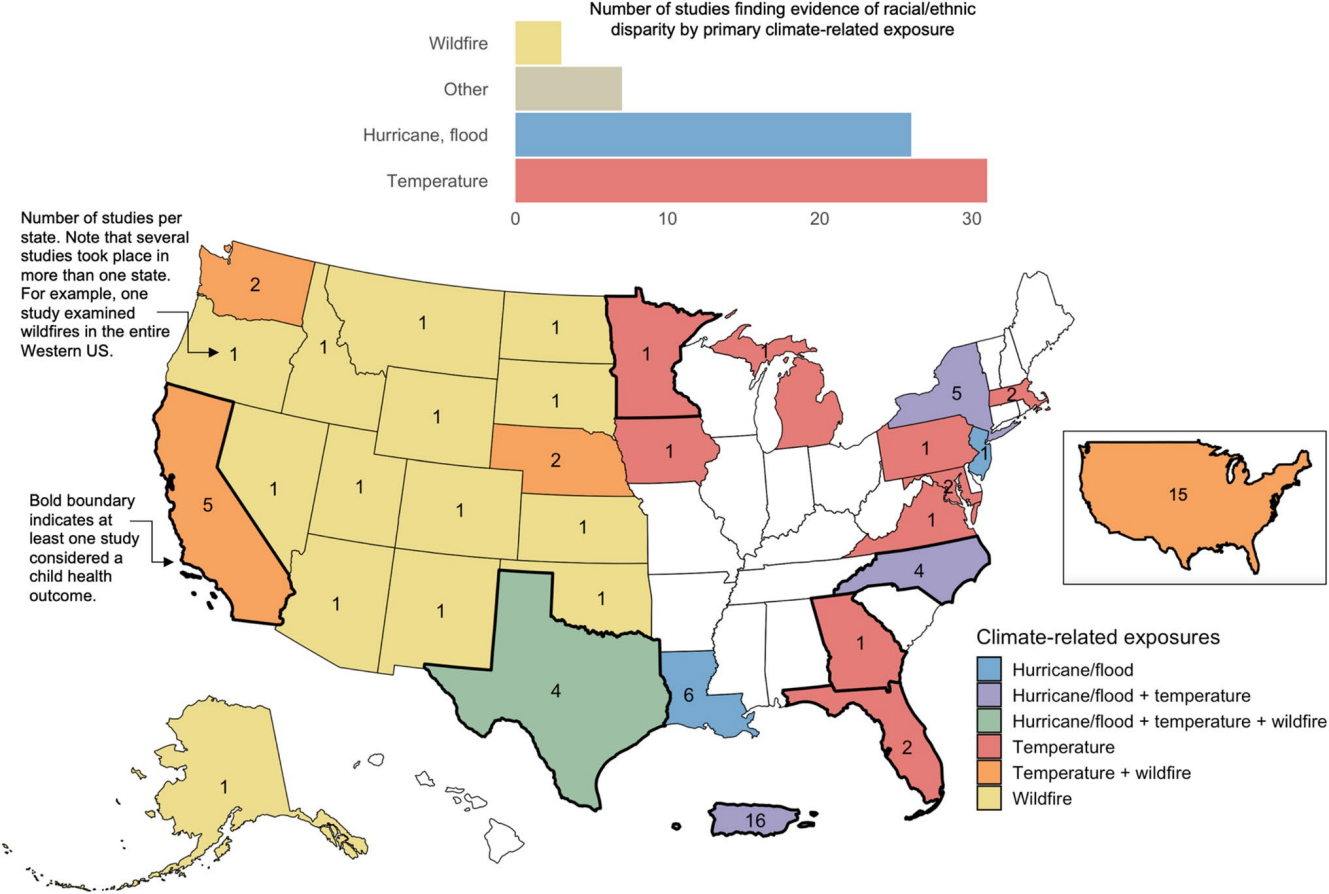
Summary of evidence of climate-related health impacts in racially marginalized communities

The impacts of temperature on racial health disparities have been examined in more than a dozen states across the country and in many nation-wide studies (Fig. 1**)**. Wildfire studies were concentrated in the Western USA, whereas studies focused on hurricanes were concentrated in the Southeast and Atlantic Coast. Racial disparities in climate-related health impacts have been much more widely studied in coastal states than in those in the interior.

### Temperature

Climate change has caused average temperatures in most parts of the USA to rise and extreme heat events to become more frequent and severe [100]. The recent literature overwhelmingly confirms earlier studies suggesting rising temperatures will lead to higher mortality and illness among adults of color than White adults. Risk of dying associated with higher temperatures and extreme heat events was elevated among Black, Latinx, and Native American individuals compared to Whites in studies across the USA, with even higher risk among non-US citizens. For example, a national study from 1999 to 2017 found that across all ages, American Indians/Native Alaskans and Blacks had higher mortality rates from excessive heat, followed by Latinos, Whites, and Asians/Pacific Islanders [71]. Another national study from 2005 to 2014 found a higher proportion of heat-related deaths among non-US citizens than US citizens. More than 85% of non-US citizens who died of excessive heat exposure in that study were Hispanic and 95% of all heat-related non-US citizen deaths took place in California, Arizona, and Texas [11]. Studies in California and Michigan found higher odds for medical clinic visits and hospitalizations due to heat-related illnesses among those identified as “non-white”, including Asians, Blacks, and Hispanics, as compared to Whites, although differences were not always statistically significant [29, 30]. Evidence from Massachusetts, Pennsylvania, Maryland, Nebraska, and New York strongly suggests Black individuals’ risk for cardiovascular, renal, and, respiratory diseases associated with heat and heatwave events exceeds that of Whites. For example, in a study of a dozen clinical sites across the country from 2002 to 2008, the authors observed higher odds of a cardiovascular event (e.g., cardiac arrest, stroke) during labor and delivery among Black (OR = 1.22, 95% CI: 1.08, 1.39) as compared to White birth parents (OR = 1.07, 95% CI: 1.02, 1.13) associated with a 1 °C increase in temperature on the day of delivery [20]. Similarly, Fisher et al. found higher odds of myocardial infarction hospitalizations associated with extreme heat events among Blacks (OR = 1.27; 95% CI: 1.12, 1.44) than Whites (OR = 1.09; 95% CI = 1.02, 1.16) in Maryland [21]. Two national studies of patients hospitalized for heat stroke observed that African Americans had the highest likelihood for acute kidney injury and rhabdomyolysis (muscle-renal syndrome) compared to Whites, Hispanics, and those categorized as “other” race [22, 23]. Finally, the odds for asthma-related ED visits were more than three times higher among Blacks than non-Blacks during a 2012 heat wave in Nebraska compared to the same period in the prior year [39].

Climate change is also contributing to colder winter weather in some parts of the country. Two national studies spanning from 1999 to 2018 found that American Indians/Alaska Natives had the highest mortality rate associated with extreme cold compared to Whites and a study in Virginia found that groups of color categorized as “non-White” were at higher risk of ED visits than Whites due to cold weather conditions [31, 71, 72••].

Workers of color are also suffering greater heat-related illness (e.g., heat rash, heat stroke, heat exhaustion) and death. Hispanics are overrepresented in industries with high heat-mortality risk including agricultural and construction [101]. Heat stress has also been found to impact dermal and pulmonary absorption of chemicals and exacerbate their toxicity, compounding the risk of illness associated with hazardous substance exposures among workers [102]. A recent study in Washington state found that Latinos were disproportionately impacted by heat-related illnesses across industries based on workers’ compensation claims from 2006 to 2017 [25]. Similarly, a national study found highest risk for heat-related death among Hispanic construction workers, particularly those born in Mexico, compared to non-Hispanic Blacks and non-Hispanic Whites [12]. Recent studies have also found elevated risk for heat-related illness among Black soldiers in the USA and Latinx farmworkers in Florida and Iowa [26–28].

Blacks and Hispanics have also been shown to experience greater adverse mental health-related outcomes (e.g., anxiety, psychosis, and substance use disorders), as measured by ED visits and self-reported symptoms, associated with temperature (heat and cold) as compared to Whites. Temperature has been linked to new onset or exacerbation of mental disorders, and people with a mental illness are also at higher risk for heat-related illness due to underlying physical pathologies (e.g., irregular thermoregulation related to schizophrenia); inability to adapt and cope with changing temperatures; or effects of psychiatric drugs on thermoregulation [103]. In California, Basu et al. found highest risk among Hispanics for mental health-related ED visits—including mental disorders, psychosis, and neurotic disorders—associated with temperature increases as compared to Asians, Blacks, and Whites from 2005 to 2013 [16]. In a study focused on two New York counties, Yoo et al. observed higher susceptibility of ED visits for mental disorders among African Americans exposed to extreme cold temperature compared to Whites [17].

While the literature overwhelmingly supports evidence for racial/ethnic disparities in temperature-related health effects, several studies that we reviewed did not find heterogeneity in effects across racial/ethnic subgroups and a small number found that the most privileged subgroup (e.g., Whites, non-Blacks) experienced higher health risks. For example, a descriptive study of heat-related illness across the USA documented a higher frequency of cases, as measured by number transported by emergency medical services, among Whites compared to American Indians/Alaska Natives, Asians, Blacks/African Americans, Hispanics/Latinx, and Native Hawaiians/Pacific Islanders [32]. A study from Nebraska found that non-African Americans had higher odds (OR = 1.85, 95% CI: 0.90, 3.80) of chronic bronchitis during a 2012 heat wave compared to a reference period in the prior year than African Americans (OR = 0.2, 95% CI: 10.01, 4.04) [40]. Yoo et al. observed higher risk for mental health-related ED visits associated with heat exposure among Whites than Black and Hispanic individuals in New York [18]. Similarly, Mason et al. observed a significantly higher proportion of Whites than Blacks or those identifying as neither Black nor White self-reporting mental health impacts related to both summer heat and winter extremes in Knoxville, Tennessee [19]. The authors of the latter study suggest that in some cases, coping behaviors and greater resilience during stressful situations (e.g., extreme weather events) may account for the relatively better mental health outcomes observed among racial minority groups.

### Hurricanes and Flooding

Climate change has been linked to more intense hurricane and precipitation events, posing threats to communities in hurricane-prone areas like the Atlantic and Gulf Coasts, Hawaii, and Puerto Rico [104]. Disproportionate flooding, less resilient infrastructure, and more limited resources for preparation and recovery among people of color can contribute to health disparities in the wake of extreme storms. Recent national-level flood risk assessments have found that economically disadvantaged populations are more likely to live in counties within flood zones and that greater flood risk is associated with higher poverty and unemployment rates [105, 106]. Studies in Texas and New York have found that flooding was significantly greater in neighborhoods with a higher proportion of non-White and socioeconomically deprived residents during Hurricanes Harvey and Sandy [107, 108].

Most studies that we reviewed focused on a few extreme hurricane events from 2005 to 2017, including Katrina, Sandy, Harvey, and Maria. The recent literature overwhelmingly suggests racial disparities in physical and mental health following hurricane events. Additionally, a growing body of literature has highlighted the health impacts and racial disparities related to natural technological (natech) disasters—cascading events in which natural disasters like floods or extreme winds trigger technological accidents that release hazardous materials. Industries utilizing hazardous materials are disproportionately located in communities of color, making them more likely to experience a natech event and increasing their risk for toxic contaminant exposure, as has been documented following Hurricanes Harvey and Katrina [109, 110].

We found strong evidence for racial/ethnic disparities in adverse mental health outcomes, including depression, anxiety, and post-traumatic stress disorder (PTSD), associated with hurricane and flooding events in studies from Louisiana, Texas, New York, and New Jersey. Black survivors of Hurricane Katrina had higher odds of screening positive for depression and increased likelihood of having PTSD compared to Whites [47, 48]. Similarly, research in the Houston metropolitan area after Hurricane Harvey found that non-Hispanic Blacks disproportionately experienced PTSD compared to non-Hispanic Whites [49]. Black and Hispanic individuals also reported higher rates of mental illness compared to Whites after Hurricane Sandy in New York and New Jersey [50].

The cardiovascular and reproductive health of people of color were also disproportionately impacted following Hurricanes Katrina and Sandy. A prospective cohort study found that Blacks had higher risk for incident cardiovascular disease associated with PTSD symptoms following Hurricane Katrina compared to Whites [56]. Becquart et al. observed exacerbation of cardiovascular disease disparities between older Black and White adults during and in the month following Hurricane Katrina [57]. Additionally, Black birth rates in New Orleans were found to decline below expected rates—as compared to a modeled counterfactual scenario—following Katrina, while White birth rates exceeded expected rates during the same period [58]. The authors attributed this decline in total Black birth rate to disproportionate out-migration by Black reproductive-aged women and changes in fertility-related behaviors (e.g., delayed childbearing for economic reasons). Finally, a New York study found that Black and Hispanic women had the highest percentage increases (20.9% and 25.4%, respectively) in pregnancy-related ED visits associated with power outages following Hurricane Sandy as compared to Whites and non-Hispanics (5.6% and 11.8%, respectively) [59].

A number of studies have focused exclusively on Puerto Rico in the aftermath of Hurricane Maria, which made landfall in September 2017 and resulted in widespread damage and a loss of power, water and basic needs for hundreds of thousands of Puerto Ricans. Many studies document a high rate of anxiety, depression, and PTSD among survivors, including those who remained on the island and those who left [51–53]. Descriptive studies of hurricane-related injuries and emergencies found that throughout the 8-week period during and following the hurricane’s landfall, ED visits for multiple causes increased by 70%, and 10% of ED visits within 6 months of Maria were due to injuries (e.g., abrasions, sprains, head injuries, concussions) sustained during the hurricane and recovery and rebuilding periods [63, 64]. Recent studies have also documented increases in chronic disease prevalence (e.g., hypertension, diabetes, obesity, and high cholesterol), declines in physical health (based on self-report), and new onset of non-communicable diseases associated with lack of hurricane preparedness, disruptions in power and water services, and changes in diet and lifestyle behaviors (e.g., sedentary activity, alcohol use, binge drinking) [60, 61]. Several studies estimated excess mortality rates attributable to Maria, with reports up to 14.3 deaths per 1,000 persons (4,645 excess deaths) from September 20 to December 31, 2017, which translates to a 62% increase as compared with the mortality rate during same period in 2016 [43–45, 46••]. Rates reported by these studies far exceed the official government death toll, suggesting a severe underestimation of hurricane-related mortality from direct and indirect causes.

A few studies of Hurricane Sandy found little evidence of health disparities or suggested greater health impacts among Whites. For example, White elderly adults in New York City experienced a higher increase in risk of cardiovascular disease, respiratory disease, and injury following the hurricane than did Blacks and those identified as “other” race [65]. Similarly, Malik et al. observed increases in overall ED utilization by older adults in the first 3 weeks following the hurricane’s landfall and, however, found a slight decrease in the proportion of Black and Hispanic patients [66]. The authors speculate that socioeconomic factors may have hindered Blacks and Hispanics from seeking ED care in the early weeks after the hurricane made landfall.

### Wildfire

Climate change is contributing to wildfires through hotter, drier weather and drought [100]. Exposure to wildfire smoke is a major health risk and can disproportionately impact sensitive groups. Recent research suggests that fine particulate matter (PM_2.5_) from wildfire smoke may be more harmful to human health than PM_2.5_ exposure from other sources (e.g., traffic emissions, power plants) [111]. Studies looking at the Western USA, Alaska, and nationally strongly suggest people of color are at higher risk for wildfire-related cardiovascular and respiratory illnesses than Whites. Specifically, a retrospective study among Medicare enrollees (aged 65 +) in 561 Western US counties found that Blacks had higher risk for respiratory hospitalization, including chronic obstructive pulmonary disease and respiratory tract infections, associated with wildfire-related PM_2.5_ on smoke-wave days (wildfire-specific PM_2.5_ concentrations above 37 μg/m^3^) vs. non-smoke-wave days compared with Whites or persons of other races [68••]. Hahn et al. found similar disparity trends during the 2015–2019 wildfire seasons in Alaska, with higher odds of asthma-related ED visits on the day of wildfire smoke PM_2.5_ exposure (defined as daily PM_2.5_ concentrations at least one standard deviation above the long-term monthly mean) and heart failure-related ED visits 5 days after exposure among Alaska Natives compared to non-Alaska Natives [69]. Finally, a retrospective study of intensive care unit admissions following exposure to wildfire-related PM_2.5_ across the USA from 2006 to 2015 found increased risk among those identified as “other” race, as compared to African Americans and Whites [70]. However, the authors note that African Americans had the smallest sample size among all groups, limiting statistical power to detect a disparate effect for this group.

### Children’s Health Outcomes

**Fig. 2:**
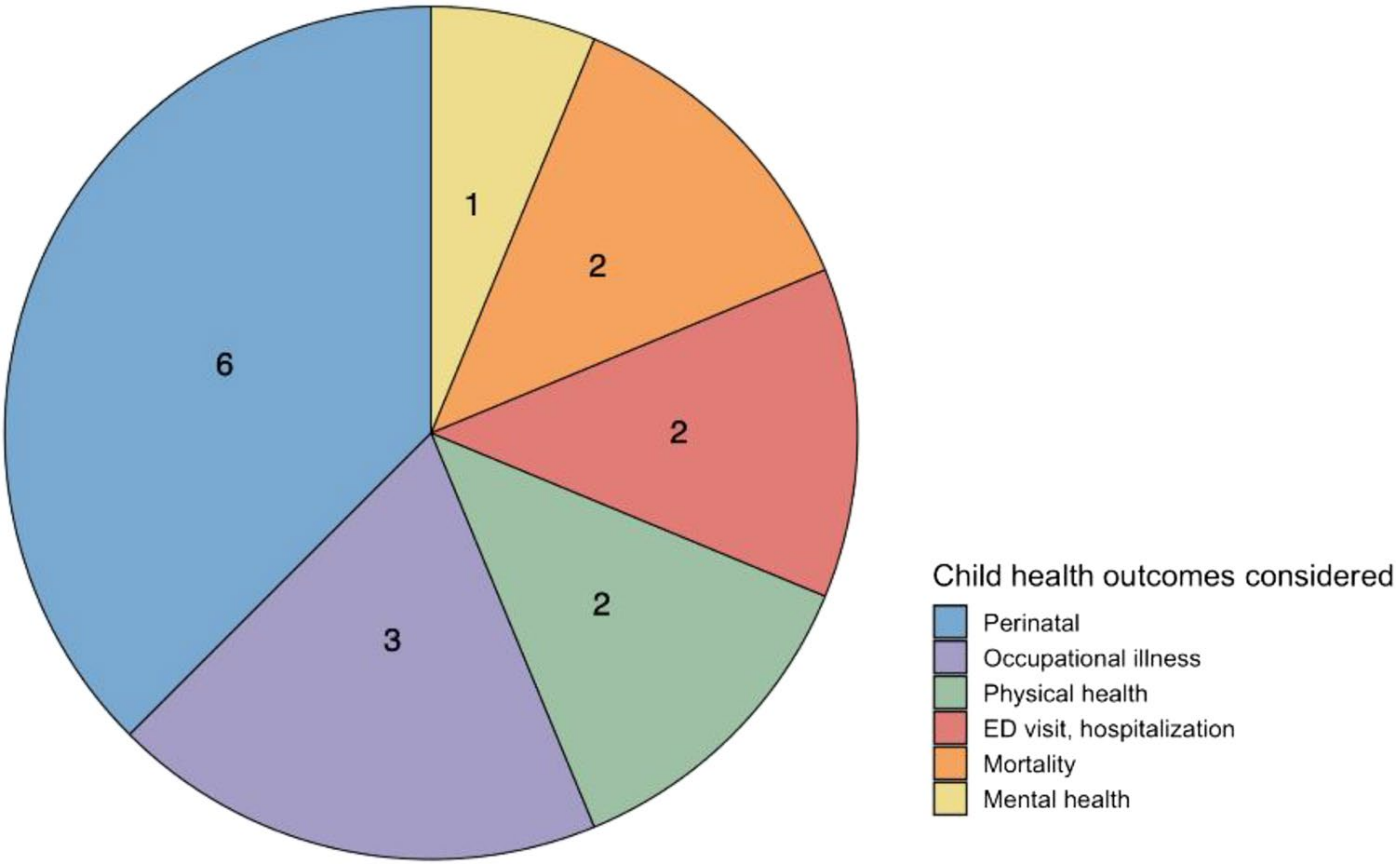
The number of studies documenting racial/ethnic disparities in children’s health associated with climatic changes by health outcome

Climate change is of particular concern for children, who have been shown in prior studies to be particularly vulnerability to climate-related health impacts like allergies, asthma, infectious diseases, PTSD, malnutrition, and poor perinatal outcomes. Children are more vulnerable due to the fact that they are still developing cognitively, physically, and physiologically and have limited ability to care for themselves [112, 113]. In the USA, children of color and in low-income families face disproportionate health risks linked to environmental exposures and social and economic stressors like housing conditions, healthcare access, and food insecurity. These early-life health disparities can result in greater chronic disease burden in adulthood. Recent studies from Puerto Rico and a number of states across the country have found that children, infants, and neonates of color experienced disproportionate health impacts associated with climate-related exposures compared to Whites (Fig. 2).

Infant mortality and adverse perinatal and childhood outcomes linked to temperature increases and extreme heat events have been shown to disproportionately impact children of color relative to Whites in national studies and studies from California, Florida, Minnesota, and Texas. Black infants’ risk for sudden infant death syndrome (SIDS) associated with a 10°F increase in temperature on the same day exceeded that of Whites (18.5%, 95% CI: 9.3, 28.5% vs. 3.6%, 95% CI: − 2.3%, 9.9%) in a study of 60,364 SIDS cases from 1972 to 2006 in 210 US cities [85]. Risk for stillbirth associated with increase in temperature was greater among non-Hispanic Blacks and Hispanics than Whites in a Florida case-crossover study of 1,876 stillbirths from 2012 to 2017 [86]. A large cohort study in California examining effects of heat on birth outcomes found that Blacks and Hispanics had the greatest risk for preterm birth (PTB, birth prior to 37 completed weeks) (24.60%, CI: 1.00, 55.27% and 17.35%, CI: 3.04, 34.98%, respectively) per 10°F increase in temperature compared to Asians (7.25%, CI: − 11.31, 30.99) and Whites (7.25%, CI: − 6.77, 22.14%) [78]. Another California cohort study found that Blacks had higher odds of term low birth weight (LBW, born after 37 completed weeks weighing less than 2,500 g) than Asians, Hispanics, and Whites [79••]. Similar trends were seen in the Midwest: a retrospective study in the Minneapolis–St. Paul metro area found that American Indians/Alaska Natives (RR = 1.31, 95% CI: 1.18, 1.46), Asians (RR = 1.07, CI: 1.00, 1.13), and Blacks (RR = 1.21, 95% CI: 1.15, 1.27) had higher risk of PTB associated with exposure to a heat wave compared to Hispanics and Whites [80]. A large retrospective birth cohort study across 50 US metropolitan areas found greater likelihood for PTB associated with heat exposure (≥ 4 consecutive days of heat in the week before delivery) among non-Hispanic Blacks and Hispanics compared to non-Hispanic Whites [81]. A retrospective cohort study in the Houston metropolitan area from 2007 to 2011 found greater risks of PTB due to extreme heat in segregated neighborhoods of concentrated racialized economic disadvantage [82]. Finally, risk of ED visits for multiple causes (e.g., ear infection, metabolic and endocrine diseases, behavioral and mental disorders) associated with extreme heat days was higher among racial minority children (RR = 1.21, 95% CI: 1.15, 1.28) than Whites (RR = 1.12; 95% CI: 1.05, 1.19) in a study of 47 children’s hospitals across the country from 2016 to 2018 [88•].

Studies in Puerto Rico have documented evidence of increased risk for adverse birth outcomes as well as physical and mental health impacts among children following hurricane events. Prior research suggests that hurricane-related stresses experienced during pregnancy (e.g., fear, property damage/loss, displacement) can increase risk for adverse birth outcomes [114]. A study of births from 1994 to 2012 in Ponce, San Juan, and Mayagüez, Puerto Rico, found strong associations between storm and flood event frequency and elevated PTB risk [98]. Infants of birth parents who experienced food insecurity for at least one month during pregnancy following Hurricane Maria had relative reductions in a specific gut bacteria, suggesting that prenatal exposure to extreme climate events can adversely impact the early-life microbiome [96]. A survey-based study among students grades 3 to 12 in all Puerto Rico public schools found that 7.2% of children (*n* = 6,900) reported clinically significant symptoms of PTSD 5 to 9 months after Hurricane Maria [95]. This study provides further support for the growing recognition of climate change-related hazards as determinants of adolescent and child mental health as they interact with and contribute to other important social determinants like education, poverty, and housing [115].

Several studies that we reviewed documented child farmworkers’ heightened risk of heat-related illnesses in an occupational setting. The agricultural industry in the USA is unique in that it allows children aged 10 and older to legally work on farms. In addition to elevated risk of heat-related illnesses, child farmworkers face risks related to occupational injuries and exposure to toxic pesticides [116]. The Child Farmworker Study, comprised of 202 Latinx child farmworkers aged 10–17 in North Carolina, is a community-based participatory research study examining the health and development of Latinx child farmworkers, almost 20% of whom are migrant workers [117]. Several descriptive studies of the children in this cohort found that nearly half experienced at least one heat-related illness while working over the preceding 12 months, a proportion that is likely to increase with climate change [92•, 93]. Symptoms included dizziness, sudden muscle cramps, hot/dry skin, nausea or vomiting, confusion, and fainting.

While the recent research strongly supports evidence for racial/ethnic disparities in climate-related health effects among children, several studies did not find heterogeneity in effects across racial/ethnic strata or found that the most privileged strata demonstrated higher health risks. For example, a study in Detroit, Michigan, found that the risk of PTB associated with high temperatures was lower among Black mothers than Whites and a study in North Carolina did not find heterogeneity in PTB risk associated with heat exposure between non-Hispanic Whites and non-Hispanic Blacks [83, 84]. A study in Philadelphia reported no effect measure modification by maternal race/ethnicity in the association between infant mortality and increased ambient temperature [87]. Finally, a California study found no difference in PTB risk across racial/ethnic groups associated with wildfire PM_2.5_ smoke exposure during pregnancy [99]. The authors note that in their sample, the burden of exposure to wildfire smoke was greatest among White mothers residing in zip-codes in the bottom 40 percentile of income, while low-income minority communities were disproportionately exposed to PM_2*.*5_ from all sources.

## Conclusion

It is clear from the recent literature that in many communities across the USA, climatic changes linked to global warming are having a disproportionate effect on the health of adults and children of color. While the findings are not universal and differences exist across racial and ethnic groups, the evidence overwhelmingly suggests climate change is an environmental injustice likely to exacerbate existing racialized health inequities in many contexts. Communities of color often face disproportionate health risks linked to cumulative exposures to environmental hazards (e.g., air pollution, traffic, contaminated water) and may be more vulnerable to the health effects associated with climate-related impacts due to racialized health and socioeconomic disparities unrelated to climate, such as systematic disinvestment in access to quality housing, education, and food. Given the unique vulnerability of children and the lifelong consequences of adverse early life experiences, more studies to understand the unequal health burden due to climate change among children of color are needed. Further work is urgently needed to examine the mechanisms by which systemic racism leads to health inequities among children and adults [118]. Several racial/ethnic groups also remain understudied. For example, we found less evidence of climate-related disparities among Asians; however, most studies combine multiple ethnicities (e.g., Filipino, Vietnamese, East Indian, Chinese, Korean, etc.) into one category, likely obscuring important differences and possible disparities across groups that have experienced marginalization differently in the USA. Disaggregation across racialized groups, such as the Asian and Latinx populations, could help researchers and policymakers identify at-risk populations. Few studies have examined the health impacts of climate change among Native Americans despite the unique cultural significance of local ecosystems and the subsistence lifestyles practiced by many Native people. Finally, the number of studies documenting disparate health impacts from climate change among Puerto Ricans and non-US citizens speak to the lack of political and civil rights as key sources of vulnerability. Future work is needed to understand the unique vulnerabilities among the undocumented immigrant population and residents of US territories besides Puerto Rico. Such an evidence base could inform climate mitigation and adaptation measures that address the root causes of climate-related health disparities in order to increase resilience among communities of color and protect children’s health.

## Supplementary Information

Below is the link to the electronic supplementary material.

Supplementary file1 (DOCX 14 KB)

## References

[1] Shepard PM, Corbin-Mark C. Climate justice. Environmental Justice. Mary Ann Liebert, Inc., publishers; 2009;2:163–6.

[2] Gutierrez KS, LePrevost CE (2016). Climate Justice in rural Southeastern United States: a review of climate change impacts and effects on human health. Int J Environ Res Public Health.

[3] Flores AB, Collins TW, Grineski SE, Griego AL, Mullen C, Nadybal SM, et al. Environmental injustice in the disaster cycle: Hurricane Harvey and the Texas Gulf Coast. Environmental Justice. Mary Ann Liebert, Inc., publishers; 2021;14:146–58.

[4] Fuller MG, Cavanaugh N, Green S, Duderstadt K. Climate change and state of the science for children’s health and environmental health equity. J Pediatr Health Care. United States; 2021. 10.1016/j.pedhc.2021.08.003 34493406

[5] Shonkoff SB, Morello-Frosch R, Pastor M, Sadd J (2011). The climate gap: environmental health and equity implications of climate change and mitigation policies in California—a review of the literature. Clim Change.

[6] Ebi KL, Ogden NH, Semenza JC, Woodward A. Detecting and attributing health burdens to climate change. Environmental Health Perspectives. Environ Health Perspect. 2017;125:085004. 10.1289/EHP1509 PMC5783629 28796635

[7] Sonali P, Nagesh KD (2020). Review of recent advances in climate change detection and attribution studies: a large-scale hydroclimatological perspective. J Water Clim Chang.

[8] Braveman P (2006). Health Disparities and health equity: concepts and measurement. Annu Rev Public Health.

[9] Ward JB, Gartner DR, Keyes KM, Fliss MD, McClure ES, Robinson WR (2019). How do we assess a racial disparity in health? Distribution, interaction, and interpretation in epidemiological studies. Ann Epidemiol.

[10] Smith KR, Woodward A, Campbell-Lendrum D, Chadee DD, Honda Y, Liu Q, Olwoch JM, Revich B, Sauerborn R. Human health: impacts, adaptation, and co-benefits. In: Climate Change 2014: Impacts, Adaptation, and Vulnerability. Part A: Global and Sectoral Aspects. Contribution of Working Group II to the Fifth Assessment Report of the Intergovernmental Panel on Climate Change. Cambridge University Press, Cambridge, United Kingdom and New York, NY, USA; 2014;709–754.

[11] Taylor EV, Vaidyanathan A, Flanders WD, Murphy M, Spencer M, Noe RS (2018). Differences in heat-related mortality by citizenship status: United States, 2005–2014. Am J Public Health.

[12] Dong XS, West GH, Holloway-Beth A, Wang X, Sokas RK (2019). Heat-related deaths among construction workers in the United States. Am J Ind Med.

[13] Li Y, Akkus C, Yu X, Joyner A, Kmet J, Sweat D, et al. Heatwave events and mortality outcomes in memphis, tennessee: testing effect modification by socioeconomic status and urbanicity. Int J Environ Res Public Health. 2019;16. 10.3390/ijerph16224568 PMC6888315 31752218

[14] Méndez-Lázaro PA, Pérez-Cardona CM, Rodríguez E, Martínez O, Taboas M, Bocanegra A (2018). Climate change, heat, and mortality in the tropical urban area of San Juan, Puerto Rico. Int J Biometeorol.

[15] Zhang Y, Xiang Q, Yu Y, Zhan Z, Hu K, Ding Z (2019). Socio-geographic disparity in cardiorespiratory mortality burden attributable to ambient temperature in the United States. Environ Sci Pollut Res Int.

[16] Basu R, Gavin L, Pearson D, Ebisu K, Malig B (2018). Examining the association between apparent temperature and mental health-related emergency room visits in California. Am J Epidemiol.

[17] Yoo E-H, Eum Y, Gao Q, Chen K (2021). Effect of extreme temperatures on daily emergency room visits for mental disorders. Environ Sci Pollut Res Int.

[18] Yoo E-H, Eum Y, Roberts JE, Gao Q, Chen K. Association between extreme temperatures and emergency room visits related to mental disorders: A multi-region time-series study in New York, USA. Sci Total Environ. Netherlands; 2021;792:148246. 10.1016/j.scitotenv.2021.148246 34144243

[19] Mason LR, Sharma BB, Walters JE, Ekenga CC. Mental Health and weather extremes in a Southeastern U.S. city: exploring group differences by race. Int J Environ Res Public Health. 2020;17. 10.3390/ijerph17103411 PMC7277714 32422909

[20] Ha S, Nguyen K, Liu D, Männistö T, Nobles C, Sherman S (2017). Ambient temperature and risk of cardiovascular events at labor and delivery: a case-crossover study. Environ Res.

[21] Fisher JA, Jiang C, Soneja SI, Mitchell C, Puett RC, Sapkota A. Summertime extreme heat events and increased risk of acute myocardial infarction hospitalizations. J Expo Sci Environ Epidemiol. United States; 2017;27:276–80. 10.1038/jes.2016.83 28176761

[22] Thongprayoon C, Qureshi F, Petnak T, Cheungpasitporn W, Chewcharat A, Cato LD, et al. Impact of acute kidney injury on outcomes of hospitalizations for heat stroke in the United States. Diseases. 2020;8. 10.3390/diseases8030028 PMC7563434 32679822

[23] Thongprayoon C, Petnak T, Kanduri SR, Kovvuru K, Cheungpasitporn W, Boonpheng B, et al. Impact of rhabdomyolysis on outcomes of hospitalizations for heat stroke in the United States. Hosp Pract (1995). England; 2020;48:276–81. 10.1080/21548331.2020.1792214 32633161

[24] Remigio RV, Jiang C, Raimann J, Kotanko P, Usvyat L, Maddux FW (2019). Association of extreme heat events with hospital admission or mortality among patients with end-stage renal disease. JAMA Netw Open.

[25] Hesketh M, Wuellner S, Robinson A, Adams D, Smith C, Bonauto D (2020). Heat related illness among workers in Washington State: a descriptive study using workers’ compensation claims, 2006–2017. Am J Ind Med.

[26] Nelson DA, Deuster PA, O’Connor FG, Kurina LM (2018). Sickle Cell Trait and Heat Injury Among US Army Soldiers. Am J Epidemiol.

[27] Mutic AD, Mix JM, Elon L, Mutic NJ, Economos J, Flocks J (2018). Classification of heat-related illness symptoms among Florida farmworkers. J Nurs Scholarsh.

[28] Culp K, Tonelli S (2019). Heat-Related illness in Midwestern Hispanic farmworkers: a descriptive analysis of hydration status and reported symptoms. Workplace Health Saf.

[29] Vashishtha D, Sieber W, Hailey B, Guirguis K, Gershunov A, Al-Delaimy WK (2018). Outpatient clinic visits during heat waves: findings from a large family medicine clinical database. Fam Pract.

[30] Ogbomo AS, Gronlund CJ, O’Neill MS, Konen T, Cameron L, Wahl R (2017). Vulnerability to extreme-heat-associated hospitalization in three counties in Michigan, USA, 2000–2009. Int J Biometeorol.

[31] Davis RE, Markle ES, Windoloski S, Houck ME, Enfield KB, Kang H (2020). A comparison of the effect of weather and climate on emergency department visitation in Roanoke and Charlottesville, Virginia. Environ Res.

[32] Yeargin S, Hirschhorn R, Grundstein A. Heat-related illnesses transported by United States Emergency Medical Services. Medicina (Kaunas). 2020;56. 10.3390/medicina56100543 PMC7602997 33080867

[33] Davis RE, Novicoff WM. The impact of heat waves on emergency department admissions in Charlottesville, Virginia, U.S.A. Int J Environ Res Public Health. 2018;15. 10.3390/ijerph15071436 PMC6068980 29986505

[34] Guirguis K, Basu R, Al-Delaimy WK, Benmarhnia T, Clemesha RES, Corcos I (2018). Heat, Disparities, and health outcomes in San Diego County’s diverse climate zones. Geohealth.

[35] Jung J, Uejio CK, Adeyeye TE, Kintziger KW, Duclos C, Reid K, et al. Using social security number to identify sub-populations vulnerable to the health impacts from extreme heat in Florida, U.S. Environ Res. Netherlands; 2021;202:111738. 10.1016/j.envres.2021.111738 34331925

[36] Schwarz L, Castillo EM, Chan TC, Brennan JJ, Sbiroli ES, Carrasco-Escobar G (2022). Heat Waves and emergency department visits among the homeless, San Diego, 2012–2019. Am J Public Health.

[37] Wang C, Solís P, Villa L, Khare N, Wentz EA, Gettel A (2021). Spatial modeling and analysis of heat-related morbidity in Maricopa County, Arizona. J Urban Health.

[38] Wei Y, Wang Y, Lin C-K, Yin K, Yang J, Shi L (2019). Associations between seasonal temperature and dementia-associated hospitalizations in New England. Environ Int.

[39] Figgs LW. Emergency department asthma diagnosis risk associated with the 2012 heat wave and drought in Douglas County NE, USA. Heart Lung. United States; 2019;48:250–7. 10.1016/j.hrtlng.2018.12.005 30686617

[40] Figgs LW (2020). Elevated chronic bronchitis diagnosis risk among women in a local emergency department patient population associated with the 2012 heatwave and drought in Douglas county. NE USA Heart Lung United States.

[41] Lane MA, Walawender M, Brownsword EA, Pu S, Saikawa E, Kraft CS, et al. The impact of cold weather on respiratory morbidity at Emory Healthcare in Atlanta. Sci Total Environ. Netherlands; 2021;813:152612. 10.1016/j.scitotenv.2021.152612 34963597

[42] Upperman CR, Parker JD, Akinbami LJ, Jiang C, He X, Murtugudde R (2017). Exposure to extreme heat events is associated with increased hay fever prevalence among nationally representative sample of US Adults: 1997–2013. J Allergy Clin Immunol Pract.

[43] Cruz-Cano R, Mead EL (2019). Causes of excess deaths in Puerto Rico after Hurricane Maria: a time-series estimation. Am J Public Health.

[44] Santos-Burgoa C, Sandberg J, Suárez E, Goldman-Hawes A, Zeger S, Garcia-Meza A (2018). Differential and persistent risk of excess mortality from Hurricane Maria in Puerto Rico: a time-series analysis. Lancet Planet Health Netherlands.

[45] Santos-Lozada AR, Howard JT (2018). Use of death counts from vital statistics to calculate excess deaths in Puerto Rico following Hurricane Maria. JAMA United States.

[46.••] Kishore N, Marqués D, Mahmud A, Kiang MV, Rodriguez I, Fuller A, et al. Mortality in Puerto Rico after Hurricane Maria. N Engl J Med. United States; 2018;379:162–70. **Household-based survey estimates that the number of excess deaths from Hurricane Maria in Puerto Rico is more than 70 times higher than the official death toll.** 10.1056/NEJMsa1803972 29809109

[47] Ali JS, Farrell AS, Alexander AC, Forde DR, Stockton M, Ward KD (2017). Race differences in depression vulnerability following Hurricane Katrina. Psychol Trauma.

[48] Alexander AC, Ali J, McDevitt-Murphy ME, Forde DR, Stockton M, Read M (2017). Racial differences in posttraumatic stress disorder vulnerability following Hurricane Katrina among a sample of adult cigarette smokers from New Orleans. J Racial Ethn Health Disparities.

[49] Flores AB, Collins TW, Grineski SE, Chakraborty J (2020). Disparities in health effects and access to health care among Houston Area residents after Hurricane Harvey. Public Health Rep.

[50] Ma C, Smith TE, Iversen RR. Mental Illness prevalence and disparities among Hurricane Sandy Survivors: a 2-year retrospective. Disaster Med Public Health Prep. United States; 2020;1–10. 10.1017/dmp.2020.46 32340644

[51] Rodriguez-Rabassa M, Hernandez R, Rodriguez Z, Colon-Echevarria CB, Maldonado L, Tollinchi N (2020). Impact of a natural disaster on access to care and biopsychosocial outcomes among Hispanic/Latino cancer survivors. Sci Rep.

[52] Carl Y, Frias RL, Kurtevski S, González Copo T, Mustafa AR, Font CM, et al. The Correlation of english language proficiency and indices of stress and anxiety in migrants from Puerto Rico after Hurricane Maria: a preliminary study. Disaster Med Public Health Prep. United States; 2020;14:23–7. 10.1017/dmp.2019.22 31221231

[53] Scaramutti C, Salas-Wright CP, Vos SR, Schwartz SJ. The mental health impact of Hurricane Maria on Puerto Ricans in Puerto Rico and Florida. Disaster Med Public Health Prep. United States; 2019;13:24–7. 10.1017/dmp.2018.151 30696508

[54] Schneider S, Rasul R, Liu B, Corry D, Lieberman-Cribbin W, Watson A (2019). Examining posttraumatic growth and mental health difficulties in the aftermath of Hurricane Sandy. Psychol Trauma.

[55] Zhang M, VanLandingham M, Park YS, Anglewicz P, Abramson DM (2021). Differences in post-disaster mental health among Vietnamese and African Americans living in adjacent urban communities flooded by Katrina. PLoS ONE.

[56] Lenane Z, Peacock E, Joyce C, Frohlich ED, Re RN, Muntner P (2019). Association of Post-Traumatic Stress Disorder Symptoms Following Hurricane Katrina With Incident Cardiovascular Disease Events Among Older Adults With Hypertension. Am J Geriatr Psychiatry.

[57] Becquart NA, Naumova EN, Singh G, Chui KKH. Cardiovascular Disease hospitalizations in Louisiana Parishes’ elderly before, during and after Hurricane Katrina. Int J Environ Res. Public Health. 2018;16. 10.3390/ijerph16010074 PMC6339087 30597886

[58] Seltzer N, Nobles J (2017). Post-Disaster fertility: hurricane katrina and the changing racial composition of New Orleans. Popul Environ.

[59] Xiao J, Zhang W, Huang M, Lu Y, Lawrence WR, Lin Z, et al. Increased risk of multiple pregnancy complications following large-scale power outages during Hurricane Sandy in New York State. Sci Total Environ. Netherlands; 2021;770:145359. 10.1016/j.scitotenv.2021.145359 PMC11875008 33736412

[60] Mattei J, Tamez M, O’Neill J, Haneuse S, Mendoza S, Orozco J (2022). Chronic diseases and associated risk factors among adults in Puerto Rico After Hurricane Maria. JAMA Netw Open.

[61] Joshipura KJ, Martínez-Lozano M, Ríos-Jiménez PI, Camacho-Monclova DM, Noboa-Ramos C, Alvarado-González GA, et al. Preparedness, Hurricanes Irma and Maria, and Impact on health in Puerto Rico. Int J Disaster Risk Reduct. 2022;67. 10.1016/j.ijdrr.2021.102657 PMC8754401 35036300

[62] Quist AJL, Fliss MD, Wade TJ, Delamater PL, Richardson DB, Engel LS (2022). Hurricane flooding and acute gastrointestinal illness in North Carolina. Sci Total Environ.

[63] Wong PW, Parton HB. Monitoring emergency department visits from Puerto Rico in the aftermath of Hurricane Maria Using syndromic surveillance - New York City, 2017. Disaster Med Public Health Prep. United States; 2020;14:44–8. 10.1017/dmp.2019.102 31642419

[64] Frasqueri-Quintana VM, Oliveras García CA, Adams LE, Torres-Figueroa X, Iriarte RI, Ryff K (2020). Injury-related emergency department visits after Hurricane Maria in a Southern Puerto Rico hospital. Disaster Med Public Health Prep.

[65] Lawrence WR, Lin Z, Lipton EA, Birkhead G, Primeau M, Dong G-H, et al. After the storm: short-term and long-term health effects following Superstorm Sandy among the elderly. Disaster Med Public Health Prep. United States; 2019;13:28–32. 10.1017/dmp.2018.152 PMC12150664 30841951

[66] Malik S, Lee DC, Doran KM, Grudzen CR, Worthing J, Portelli I, et al. Vulnerability of older adults in disasters: emergency department utilization by geriatric patients after Hurricane Sandy. Disaster Med Public Health Prep. United States; 2018;12:184–93. 10.1017/dmp.2017.44 28766475

[67] Cruz NI, Santiago E, Rodríguez K. The Effect of hurricane maria on the surgical workload of the UPR-affiliated hospitals. P R Health Sci J. Puerto Rico; 2020;39:195–9. 32663917

[68.••] Liu JC, Wilson A, Mickley LJ, Ebisu K, Sulprizio MP, Wang Y, et al. Who Among the Elderly is most vulnerable to exposure to and health risks of fine particulate matter from wildfire smoke? Am J Epidemiol. 2017;186:730–5. **Retrospective multi-year and multi-state study on the association between wildfire-related PM**_**2.5**_** and respiratory hospitalizations uses daily source-specific exposure estimates that distinguish wildfire-specific PM**_**2.5**_** from other PM**_**2.5**_** sources.** 10.1093/aje/kwx141 PMC5860049 28525551

[69] Hahn MB, Kuiper G, O’Dell K, Fischer EV, Magzamen S. Wildfire smoke is associated with an increased risk of cardiorespiratory emergency department visits in Alaska. Geohealth. 2021;5:e2020GH000349. 10.1029/2020GH000349 PMC8137270 34036208

[70] Sorensen C, House JA, O’Dell K, Brey SJ, Ford B, Pierce JR, et al. Associations between wildfire-related PM(2.5) and Intensive care unit admissions in the United States, 2006–2015. Geohealth. 2021;5:e2021GH000385. 10.1029/2021GH000385 PMC8095362 33977181

[71] Adams RM, Evans CM, Mathews MC, Wolkin A, Peek L. Mortality from forces of nature among older adults by race/ethnicity and gender. J Appl Gerontol. United States; 2020;733464820954676. 10.1177/0733464820954676 32909520

[72.••] Sharpe JD, Wolkin AF. The epidemiology and geographic patterns of natural disaster and extreme weather mortality by race and ethnicity, United States, 1999–2018. Public Health Rep. United States; 2021;333549211047235. **National mortality data from the Centers for Disease Control and Prevention from 1999 to 2018 reveals racial disparities in age-adjusted mortality from extreme weather events and natural disasters.**

[73.••] Lynch KM, Lyles RH, Waller LA, Abadi AM, Bell JE, Gribble MO. Drought severity and all-cause mortality rates among adults in the United States: 1968–2014. Environ Health. 2020;19:52. **National multi-decade retrospective study finds heterogeneous associations between drought and all-cause mortality in subpopulation-stratified models, suggesting that impacts vary across age, race, and climate region.** 10.1186/s12940-020-00597-8 PMC7236144 32423443

[74.•] Sapkota A, Dong Y, Li L, Asrar G, Zhou Y, Li X, et al. Association between changes in timing of spring onset and asthma hospitalization in Maryland. JAMA Netw Open. 2020;3:e207551. **Cross-sectional study of 29,257 asthma patients on the association between timing of spring onset and daily asthma hospitalization highlights how climate-induced ecological changes may affect the burden of allergic diseases.** 10.1001/jamanetworkopen.2020.7551 PMC7339136 32663309

[75] Geier DA, Kern JK, Geier MR (2018). A longitudinal ecological study of seasonal influenza deaths in relation to climate conditions in the United States from 1999 through 2011. Infect Ecol Epidemiol.

[76] Smith GS, Messier KP, Crooks JL, Wade TJ, Lin CJ, Hilborn ED (2017). Extreme precipitation and emergency room visits for influenza in Massachusetts: a case-crossover analysis. Environ Health.

[77] Yue H, Hu T. Geographical Detector-Based Spatial modeling of the COVID-19 Mortality rate in the continental United States. Int J Environ Res Public Health. 2021;18. 10.3390/ijerph18136832 PMC8296863 34202168

[78] Basu R, Chen H, Li D-K, Avalos LA (2017). The impact of maternal factors on the association between temperature and preterm delivery. Environ Res.

[79.••] Basu R, Rau R, Pearson D, Malig B. Temperature and term low birth weight in California. Am J Epidemiol. United States; 2018;187:2306–14. **Multi-year retrospective study of a large, racially diverse cohort suggests evidence for adverse perinatal outcomes associated with extreme heat for vulnerable subgroups of pregnant women.** 10.1093/aje/kwy116 29901701

[80] Smith ML, Hardeman RR. Association of summer heat waves and the probability of preterm birth in Minnesota: an exploration of the intersection of race and education. Int J Environ Res Public Health. 2020;17. 10.3390/ijerph17176391 PMC7503599 32887349

[81] Huang M, Strickland MJ, Richards M, Holmes HA, Newman AJ, Garn JV (2021). Acute associations between heatwaves and preterm and early-term birth in 50 US metropolitan areas: a matched case-control study. Environ Health.

[82] Cushing L, Morello-Frosch R, Hubbard A (2022). Extreme heat and its association with social disparities in the risk of spontaneous preterm birth. Paediatr Perinat.

[83] Gronlund CJ, Yang AJ, Conlon KC, Bergmans RS, Le HQ, Batterman SA (2020). Time series analysis of total and direct associations between high temperatures and preterm births in Detroit, Michigan. BMJ Open.

[84] Son J-Y, Choi HM, Miranda ML, Bell ML (2022). Exposure to heat during pregnancy and preterm birth in North Carolina: Main effect and disparities by residential greenness, urbanicity, and socioeconomic status. Environ Res.

[85] Jhun I, Mata DA, Nordio F, Lee M, Schwartz J, Zanobetti A (2017). Ambient temperature and sudden infant death syndrome in the United States. Epidemiology.

[86] Savitz DA, Hu H. Ambient heat and stillbirth in Northern and Central Florida. Environ Res. Netherlands; 2021;199:111262. 10.1016/j.envres.2021.111262 PMC8638076 33974845

[87] Schinasi LH, Bloch JR, Melly S, Zhao Y, Moore K, De Roos AJ. High ambient temperature and infant mortality in Philadelphia, Pennsylvania: a case–crossover study. Am J Public Health. American Public Health Association; 2020;110:189–95. 10.2105/AJPH.2019.305442 PMC6951370 31855483

[88.•] Bernstein AS, Sun S, Weinberger KR, Spangler KR, Sheffield PE, Wellenius GA. Warm season and emergency department visits to U.S. children’s hospitals. environmental health perspectives. Environ Health Perspect. 2022;130:017001. **Time-series analysis of the association between extreme heat exposure and ED visits by children and adolescents leverages data on 3.8 million ED visits from 47 US children’s hospitals from 2016 to 2018.**

[89] Niu L, Herrera MT, Girma B, Liu B, Schinasi L, Clougherty JE (2022). High ambient temperature and child emergency and hospital visits in New York City. Paediatr Perinat Epidemiol.

[90] O’Lenick CR, Winquist A, Chang HH, Kramer MR, Mulholland JA, Grundstein A (2017). Evaluation of individual and area-level factors as modifiers of the association between warm-season temperature and pediatric asthma morbidity in Atlanta, GA. Environ Res.

[91] Sheffield PE, Herrera MT, Kinnee EJ, Clougherty JE (2018). Not so little differences: variation in hot weather risk to young children in New York City. Public Health.

[92.•] Arcury TA, Quandt SA, Arnold TJ, Chen H, Daniel SS. Occupational injuries of Latinx child farmworkers in north carolina: associations with work safety culture. J Occup Environ Med. United States; 2020;62:853–8. **The Hired Child Farmworker Study is a large community-based participatory research study in North Carolina comprised of migrant and non-migrant Latinx farmworkers aged 10–17 that documents child farmworkers’ occupational injuries and health characteristics.** 10.1097/JOM.0000000000001982 PMC8875827 32769794

[93] Arnold TJ, Arcury TA, Sandberg JC, Quandt SA, Talton JW, Mora DC (2020). Heat-Related Illness Among Latinx Child Farmworkers in North Carolina: a mixed-methods study. New Solut.

[94] Arcury TA, Arnold TJ, Quandt SA, Chen H, Kearney GD, Sandberg JC, et al. Health and Occupational injury experienced by Latinx child farmworkers in North Carolina, USA. Int J Environ Res Public Health. 2019;17. 10.3390/ijerph17010248 PMC6981743 31905836

[95] Orengo-Aguayo R, Stewart RW, de Arellano MA, Suárez-Kindy JL, Young J (2019). Disaster exposure and mental health among Puerto Rican Youths After Hurricane Maria. JAMA Netw Open.

[96] Wang L, de Ángel SD, Acevedo Flores M, Schriefer A, Wang L, Gerónimo López K (2020). Prenatal food insecurity post Hurricane Maria is associated with decreased Veillonella in the infant gut. Pediatr Res.

[97] Arlinghaus KR, Gorniak SL, Hernandez DC, Johnston CA. Impact of hurricane harvey on the growth of low income, ethnic minority adolescents. Disaster Med Public Health Prep. United States; 2020;1–8. 10.1017/dmp.2020.308 33161933

[98] Yu X, Feric Z, Cordero JF, Meeker JD, Alshawabkeh A (2018). Potential influence of temperature and precipitation on preterm birth rate in Puerto Rico. Sci Rep.

[99] Heft-Neal S, Driscoll A, Yang W, Shaw G, Burke M. Associations between wildfire smoke exposure during pregnancy and risk of preterm birth in California. Environ Res. Netherlands; 2022;203:111872. 10.1016/j.envres.2021.111872 34403668

[100] Romero-Lankao P, Smith JB, Davidson DJ, Diffenbaugh NS, Kinney PL, Kirshen P, Kovacs P, Villers Ruiz L. North America. In: Climate Change 2014: Impacts, adaptation, and vulnerability. Part B: Regional Aspects. Contribution of Working Group II to the Fifth Assessment Report of the Intergovernmental Panel on Climate Change. Cambridge University Press, Cambridge, United Kingdom and New York, NY, USA; 2014;1439–1498.

[101] Gubernot DM, Anderson GB, Hunting KL (2015). Characterizing occupational heat-related mortality in the United States, 2000–2010: an analysis using the Census of Fatal Occupational Injuries database. Am J Ind Med.

[102] Applebaum KM, Graham J, Gray GM, LaPuma P, McCormick SA, Northcross A (2016). An Overview of occupational risks from climate change. Curr Envir Health Rpt.

[103] Cusack L, de Crespigny C, Athanasos P (2011). Heatwaves and their impact on people with alcohol, drug and mental health conditions: a discussion paper on clinical practice considerations. J Adv Nurs.

[104] Collins M, Sutherland M, Bouwer L, Cheong SM, Frölicher T, Jacot Des Combes H, Koll Roxy M, Losada I, McInnes K, Ratter B, Rivera-Arriaga E, Susanto RD, Swingedouw D, Tibig L. Extremes, abrupt changes and managing risk. In: IPCC Special Report on the Ocean and Cryosphere in a Changing Climate. In Press.

[105] Rhubart D, Sun Y (2021). The social correlates of flood risk: variation along the US rural–urban continuum. Popul Environ.

[106] Qiang Y (2019). Disparities of population exposed to flood hazards in the United States. J Environ Manage.

[107] Chakraborty J, Collins TW, Grineski SE. Exploring the environmental justice implications of hurricane harvey flooding in Greater Houston, Texas. Am J Public Health. American Public Health Association; 2019;109:244–50. 10.2105/AJPH.2018.304846 PMC6336065 30571302

[108] Lieberman-Cribbin W, Gillezeau C, Schwartz RM, Taioli E (2021). Unequal social vulnerability to Hurricane Sandy flood exposure. J Expo Sci Environ Epidemiol.

[109] Flores AB, Castor A, Grineski SE, Collins TW, Mullen C (2021). Petrochemical releases disproportionately affected socially vulnerable populations along the Texas Gulf Coast after Hurricane Harvey. Popul Environ.

[110] Picou JS. Katrina as a Natech Disaster: Toxic Contamination and long-term risks for residents of New Orleans. J Appl Soc Sci. SAGE Publications Inc; 2009;3:39–55.

[111] Aguilera R, Corringham T, Gershunov A, Benmarhnia T (2021). Wildfire smoke impacts respiratory health more than fine particles from other sources: observational evidence from Southern California. Nat Commun.

[112] Anderko L, Chalupka S, Du M, Hauptman M (2020). Climate changes reproductive and children’s health: a review of risks, exposures, and impacts. Pediatr Res.

[113] Bunyavanich S, Landrigan CP, McMichael AJ, Epstein PR (2003). The impact of climate change on child health. Ambul Pediatr.

[114] Currie J (2013). Weathering the storm: hurricanes and birth outcomes. J Health Econ.

[115] van Nieuwenhuizen A, Hudson K, Chen X, Hwong AR (2021). The Effects of climate change on child and adolescent mental health: clinical considerations. Curr Psychiatry Rep.

[116] McLaurin JA, Liebman AK (2012). Unique agricultural safety and health issues of migrant and immigrant children. J Agromed.

[117] Arcury TA, Arnold TJ, Sandberg JC, Quandt SA, Talton JW, Malki A (2019). Latinx child farmworkers in North Carolina: study design and participant baseline characteristics. Am J Ind Med.

[118] Lett E, Asabor E, Beltrán S, Michelle Cannon A, Arah OA. Conceptualizing, Contextualizing, and operationalizing race in quantitative health sciences research. Ann Fam Med. 2022;2792. 10.1370/afm.2792 PMC8959750 35045967

