# Supplementary material for: Racial Disparities in Climate Change-Related Health Effects in the United States
Source: Curr Environ Health Rep. 2022 May 28;9(3):451–64. doi: 10.1007/s40572-022-00360-w (PMC9363288; doi:10.1007/s40572-022-00360-w)
Supplement: Supplementary file 1 — Supplementary file1 (DOCX 14 KB) [file 40572_2022_360_MOESM1_ESM.docx]

**Table S1.** PubMed search terms

| **Terms** |  | **Syntax** |
| --- | --- | --- |
| Climate change | | "climate change"[tiab] OR "climatic change"[tiab] OR "global warming"[tiab] OR "climatic processes"[MeSH] OR "extreme weather"[MeSH] OR precipitation[tiab] OR heat[tiab] OR cold[tiab] OR frost[tiab] OR flood*[tiab] OR inundat*[tiab] OR "sea level rise" [tiab] OR drought*[tiab] OR cyclone*[tiab] OR tornado*[tiab] OR (storm*[tiab] AND weather[tiab]) OR hurricane*[tiab] OR natech*[tiab] OR "na-tech"[tiab] OR wildfire*[tiab] |
| *AND* | |  |
| Health | | health[tiab] OR disease*[tiab] OR illness*[tiab] OR morbidity[tiab] OR mortality[tiab] OR death*[tiab] OR injury[tiab] OR hospital*[tiab] OR "emergency department"[tiab] OR "emergency room"[tiab] OR asthma[tiab] OR allerg*[tiab] OR birth*[tiab] OR perinatal[tiab] OR pediatric*[tiab] OR pregnancy[tiab] OR vector*[tiab] OR "water-borne"[tiab] OR "food-borne"[tiab] OR nutrition*[tiab] |
| *AND* | |  |
| Race/ethnicity, disparity | | race[tiab] OR racial*[tiab] OR ethnic*[tiab] OR indigenous[tiab] OR "Native American"[tiab] OR tribe*[tiab] OR "Alaska Native"[tiab] OR Asian*[tiab] OR "Pacific Islander"[tiab] OR API[tiab] OR "African American"[tiab] OR Black*[tiab] OR Hispanic*[tiab] OR Latin*[tiab] OR "Puerto Rico"[tiab] OR marginalized[tiab] OR disparit*[tiab] OR disproportion*[tiab] OR "climate justice"[tiab] OR "environmental justice"[tiab] OR "environmental racism"[tiab] |
